# 3D Bioprinting: Shaping the Future of Periodontal Tissue Regeneration and Disease Management

**DOI:** 10.7759/cureus.82432

**Published:** 2025-04-17

**Authors:** Jahnavi R Acharya, Santosh Kumar, Gaurav A Girdhar, Shirishkumar Patel, Nirav H Parekh, Hiren H Patadiya, Anjali Narsinhbhai Zinjala, Mainul Haque

**Affiliations:** 1 Department of Periodontology and Implantology, Karnavati School of Dentistry, Karnavati University, Gandhinagar, IND; 2 Department of Periodontology, Smile Rite Dental Care, Southington, USA; 3 Department of General Dentistry, My Dental, Southbridge, USA; 4 Department of Pharmacology and Therapeutics, National Defence University of Malaysia, Kuala Lumpur, MYS; 5 Department of Research, Karnavati School of Dentistry, Karnavati University, Gandhinagar, IND

**Keywords:** biocompatible bioinks, biomaterials, extrusion-based 3d bioprinting, growth factors, hydrogel, mesenchymal stem cells, patient-specific tissue constructs, periodontitis, regenerative periodontal tissues, tissue engineering

## Abstract

The periodontium is one of the most complex tissues in the body, consisting of a hierarchical blend of soft and hard tissues. Its complex architecture makes treating and regenerating disease-damaged periodontal tissues a persistent challenge in biomedicine. Three-dimensional (3D) bioprinting represents a transformative approach to tissue engineering, offering promising advancements in treating and regenerating periodontal disease. This innovative technology enables the precise fabrication of complex, patient-specific tissue structures, facilitating the repair and restoration of damaged periodontal tissues, including the gingiva, bone, and periodontal ligament (PDL). By utilizing biocompatible materials such as living cells, hydrogels, and growth factors, 3D bioprinting has the potential to create functional, biologically integrated constructs that can mimic the natural architecture of periodontal tissues. However, translating these advancements into clinical applications remains a challenge. Emerging technologies like bioprinting have been developed to address some limitations of traditional tissue engineering methods. This review explores the current state of 3D bioprinting technology, its application in periodontal disease treatment, and the challenges associated with scaling up this technology for clinical use. Additionally, it discusses the future implications of bioprinting for personalized medicine, offering a new frontier for regenerating periodontal tissues and improving patient outcomes in oral health. Integrating 3D bioprinting into periodontal regenerative therapies could revolutionize clinical practices, offering more effective, tailored, and sustainable solutions to address the challenges of periodontal disease.

## Introduction and background

**Figure 1:**
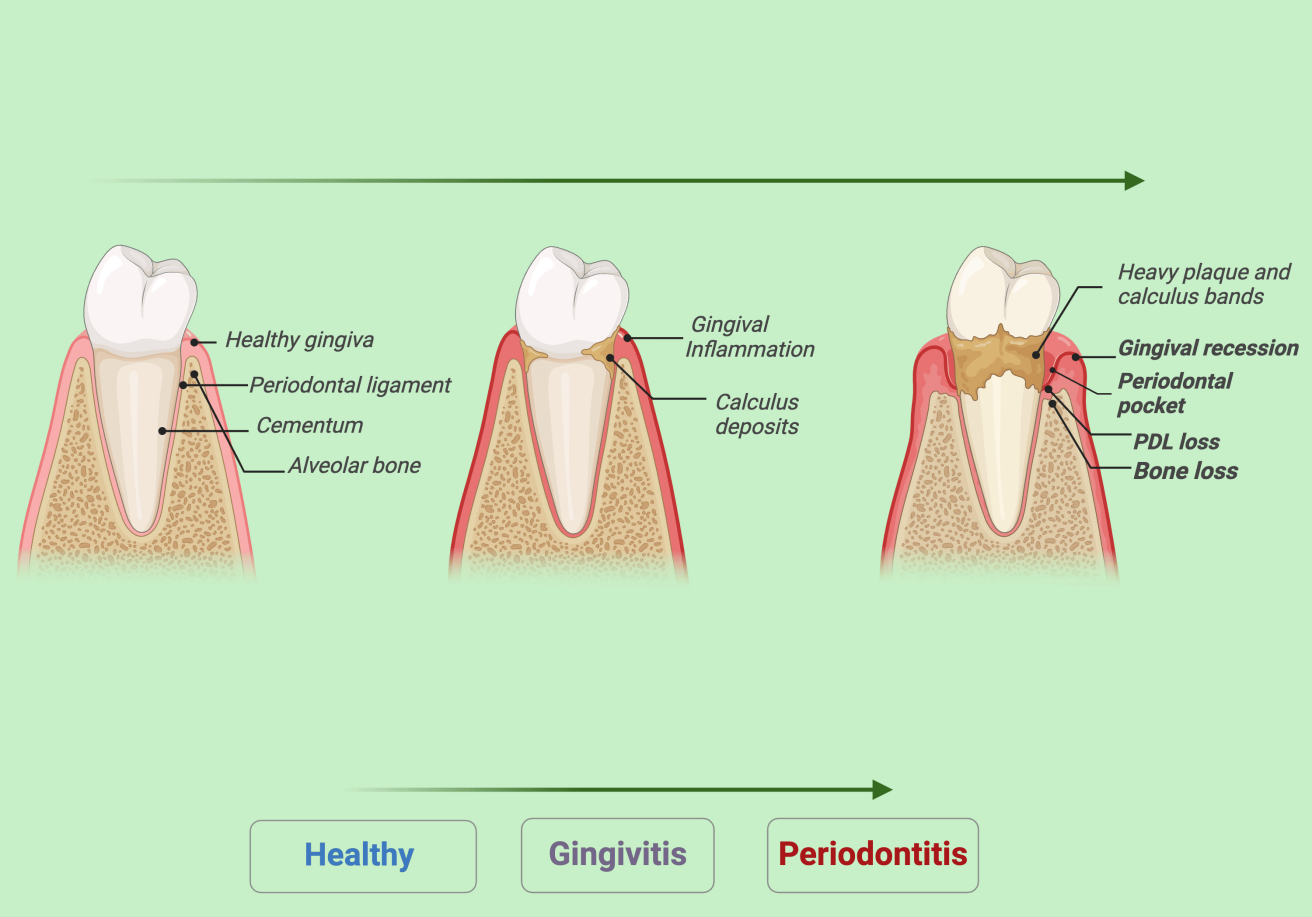
Progressive loss of periodontal tissues due to periodontitis. Notes: The progression from a healthy tooth to periodontitis begins with plaque buildup, leading to gingivitis, characterized by gum inflammation. If untreated, it advances to periodontitis, causing periodontal pockets, gingival recession, loss of the periodontal ligament (PDL), and bone loss, ultimately risking tooth loss. Illustration Credit: Dr. Jahnavi Acharya. This image was created using the premium version of BioRender [15] (https://BioRender.com/057o5), accessed on March 30, 2025, with agreement license number EL2834JV21. We did not utilize anything from the BioRender Template Library.

Oral health is essential to overall health [1-3], representing a significant health challenge in many nations [4]. The periodontium comprises the supporting tissues around the teeth, such as the cementum, periodontal ligament, gingiva, and alveolar bone (Figure 1) [5]. The periodontal ligament is a connective tissue mainly made of collagen fiber bundles [6-9]. It provides elasticity and proprioception and helps anchor the tooth. It also regenerates tissues and maintains alveolar bone homeostasis [5]. Periodontal disease is an inflammation of the supporting tissues, leading to the gradual destruction and loss of bone and periodontal ligaments [10]. Periodontal diseases, including gingivitis and periodontitis, are some of the most prevalent oral health issues worldwide, affecting a considerable proportion of the global population. These diseases primarily result in the progressive loss of periodontal tissues (Figure 1), which include the periodontal ligament (PDL), alveolar bone, and gingiva, leading to tooth mobility and, in severe cases, tooth loss [11]. Issues stemming from limited regenerative ability can impact various dentoalveolar tissues. For instance, injuries, genetic disorders, or tumors may result in defects in the alveolar bone [12]. Additionally, periodontitis, which can cause bone loss and ultimately lead to tooth loss [13], is recognized as the sixth most prevalent disease worldwide, affecting 45%-50% of the population [14].

Severe alveolar bone loss resulting from periodontitis is one of the major contributors to tooth loss in adults [10,16]. In their study, Nascimento et al. forecasted the burden of severe periodontitis and edentulism in 2021 and predicted the same for 2025 [17]. They also compared the Level-4 leading diseases/conditions based on the years lived with disability affecting humans globally in 2021 and 2050. In 2021, severe periodontitis was ranked the 31st most significant Level-4 disease/condition worldwide, and edentulism ranked 24th. It is further predicted that periodontitis and edentulism will be ranked 30th and 15th in 2050, respectively [17]. Compared to 1990, there has been a rise in the total number of globally prevalent cases (+99.96%) and new cases (+93.56%) of severe periodontitis. By 2050, it is estimated that more than 1.56 billion people will be affected by severe periodontitis, representing an increase of 500 million individuals compared to 2021 [17]. In 2021, South Asia exhibited the highest prevalence of severe periodontitis, and this pattern is expected to persist until 2050. This region encompasses three densely populated low- and middle-income countries: Bangladesh, Pakistan, and India, the world's most populous. In addition to socioeconomic factors influencing the spread of the disease [18], South Asian nations have become new focal points for the tobacco industry, as restrictions have increased in other regions [19]. Furthermore, the widespread practice of betel quid chewing in this area is linked to a higher incidence of severe periodontitis [20].

While conventional periodontal treatments such as scaling and root planing, bone grafting, and soft tissue augmentation have proven effective to a certain extent, they often fail to fully restore the damaged tissues to their original form and function [21]. Available treatments for periodontitis are time-consuming; sub-optimal in the restoration of lost tissue, affected by pre-existing conditions; and, most importantly, unsuccessful in 20%-30% of cases [22,23]. Moreover, such treatments are limited by their ability to regenerate complex periodontal structures, which require structural integrity and functional restoration [24].

Integrating advanced technologies, mainly 3D bioprinting, into periodontics has generated significant interest and research. Bioprinting, as the name suggests, refers to printing living tissues. This is accomplished with 3D bioprinters that utilize a computer-aided design (CAD) model. In this process, bio-inks are deposited in layers through an additive manufacturing technique to form tissues that replicate the structure and function of natural tissues [25]. 3D bioprinting offers the potential to create highly personalized, patient-specific tissue constructs that can replicate the native architecture of periodontal tissues. This technology allows for the precise deposition of biomaterials and living cells in a layer-by-layer fashion, enabling the construction of complex tissue structures with high accuracy [26].

**Figure 2:**
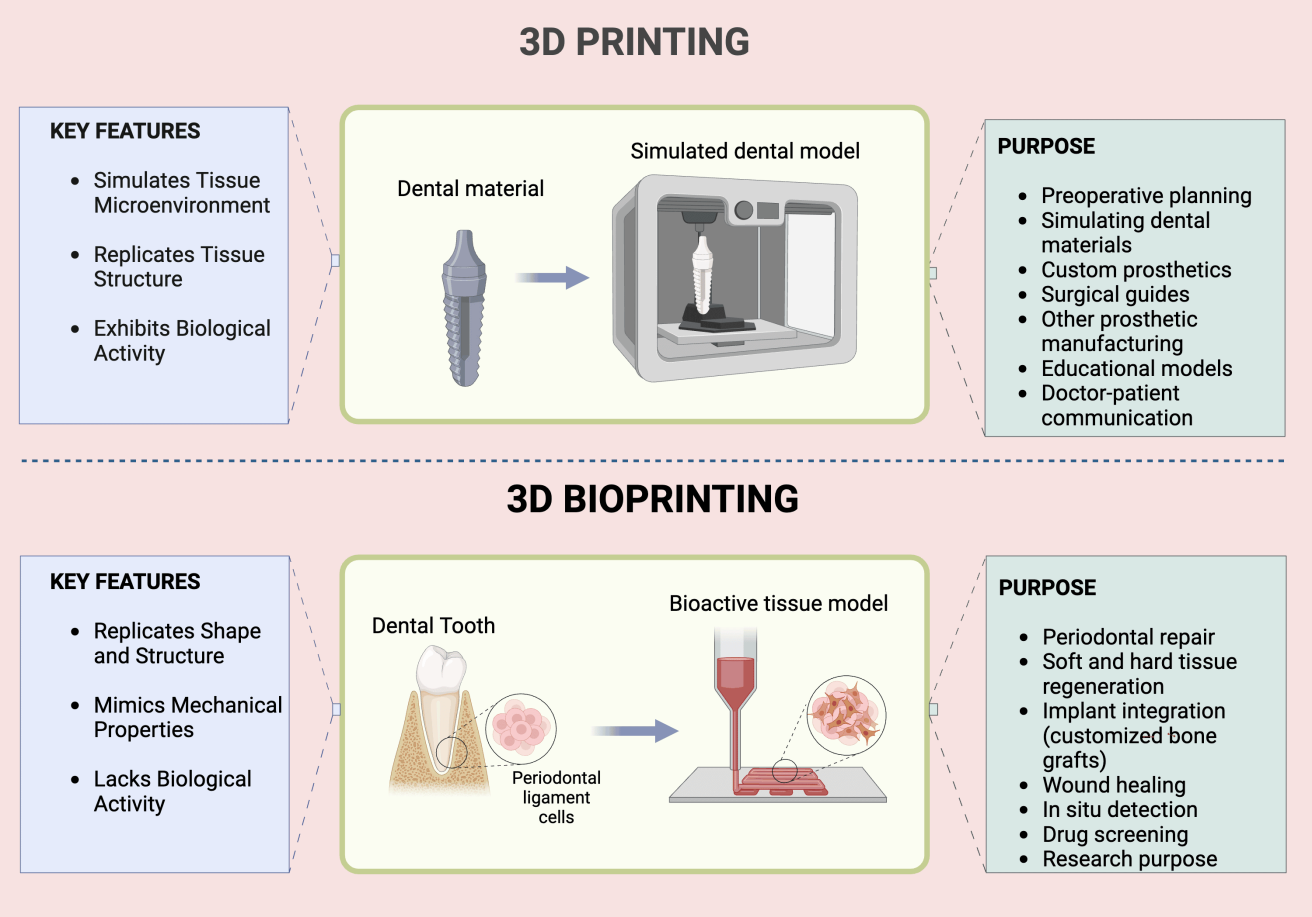
Difference between 3D printing and 3D bioprinting. Illustration Credit: Dr. Jahnavi Acharya. This image was created using the premium version of BioRender [15] (https://BioRender.com/m9icrss), accessed on March 30, 2025, with an agreement license number HJ2834WQZ4. We did not use anything from the BioRender Template Library.

It is essential to differentiate between "3D printing" and "3D bioprinting" (Figure 2), as these terms are often used interchangeably in the scientific community. While both processes involve building a 3D object layer-by-layer from a 3D model, 3D bioprinting uses cell-laden bio-inks and other biologics to create living tissues. In contrast, traditional 3D printing does not incorporate cells or biologics. For example, the 3D printing of porous polymeric scaffolds for cell seeding should not be confused with the bioprinting of cell-laden bio-inks. 3D printing has various biomedical applications, such as devices, surgical instruments, prostheses, customized implants (made from inert materials like metals, ceramics, or polymers without cells), and anatomical models for surgical planning and training. This review, however, focuses exclusively on bioprinting for regenerative periodontal applications, which involves using cell-laden bio-inks. Therefore, general 3D printing technologies and applications are outside the scope of this work [24,27].

The concept of 3D bioprinting involves not just the deposition of cells but also the careful manipulation of the microenvironment to promote cell survival, differentiation, and tissue maturation. The "triad of bioprinting" refers to the three essential components required to fabricate functional 3D tissues and organs: cells, biomaterials (or scaffolds), and signaling factors. These elements work synergistically to recreate the native tissue environment, supporting cell viability, guiding differentiation, and enabling tissue regeneration [5]. Moreover, the possibility of incorporating different cell types, such as mesenchymal stem cells (MSCs) and fibroblasts, further enhances the potential for regenerative therapy [28].

Several key challenges remain in the clinical translation of 3D bioprinting for periodontal regeneration, including better vascularization, integration with host tissues, and long-term stability of bioprinted constructs. Despite these challenges, ongoing advancements in bioprinting technologies, materials science, and tissue engineering have brought us closer to the potential clinical application of this technology. Notably, 3D bioprinting has shown promise in regenerating periodontal structures like the PDL, alveolar bone, and gingival tissues, which are essential for the long-term success of periodontal therapies [29,30].

In addition to its regenerative potential, 3D bioprinting allows the creation of customized, individualized treatment plans for patients, enabling particular tissue constructs tailored to their unique anatomical and pathological needs. This level of precision and customization represents a significant leap forward from conventional treatments, offering hope for more effective and comprehensive solutions for patients suffering from periodontal diseases [24].

Problem statement of this study

Periodontal diseases, particularly periodontitis, present significant challenges in oral health, affecting millions worldwide and leading to the progressive destruction of supporting periodontal tissues. Traditional treatment modalities, such as scaling, root planing, bone grafting, and soft tissue augmentation, often fail to fully restore the complex structure and function of the damaged periodontal tissues. Moreover, these treatments are time-consuming, less effective in restoring native tissue integrity, and unsuccessful in 20%-30% of cases. The complex architecture and multifaceted nature of periodontal tissues necessitate advanced regenerative solutions that can provide both structural and functional restoration. The emergence of 3D bioprinting offers a novel approach to address these challenges by enabling the precise fabrication of patient-specific tissue constructs. However, vascularization, tissue integration, and the long-term stability of bioprinted constructs continue to impede clinical translation. Therefore, the significance of 3D bioprinting in the field of periodontics needs to be further explored.

The objective of this study

This review examines the current advancements and applications of 3D bioprinting in periodontics. It explores the fundamental principles of this technology, the biomaterials used, the challenges faced, and its diverse applications in periodontal tissue regeneration.

## Review

Materials and methods

**Figure 3:**
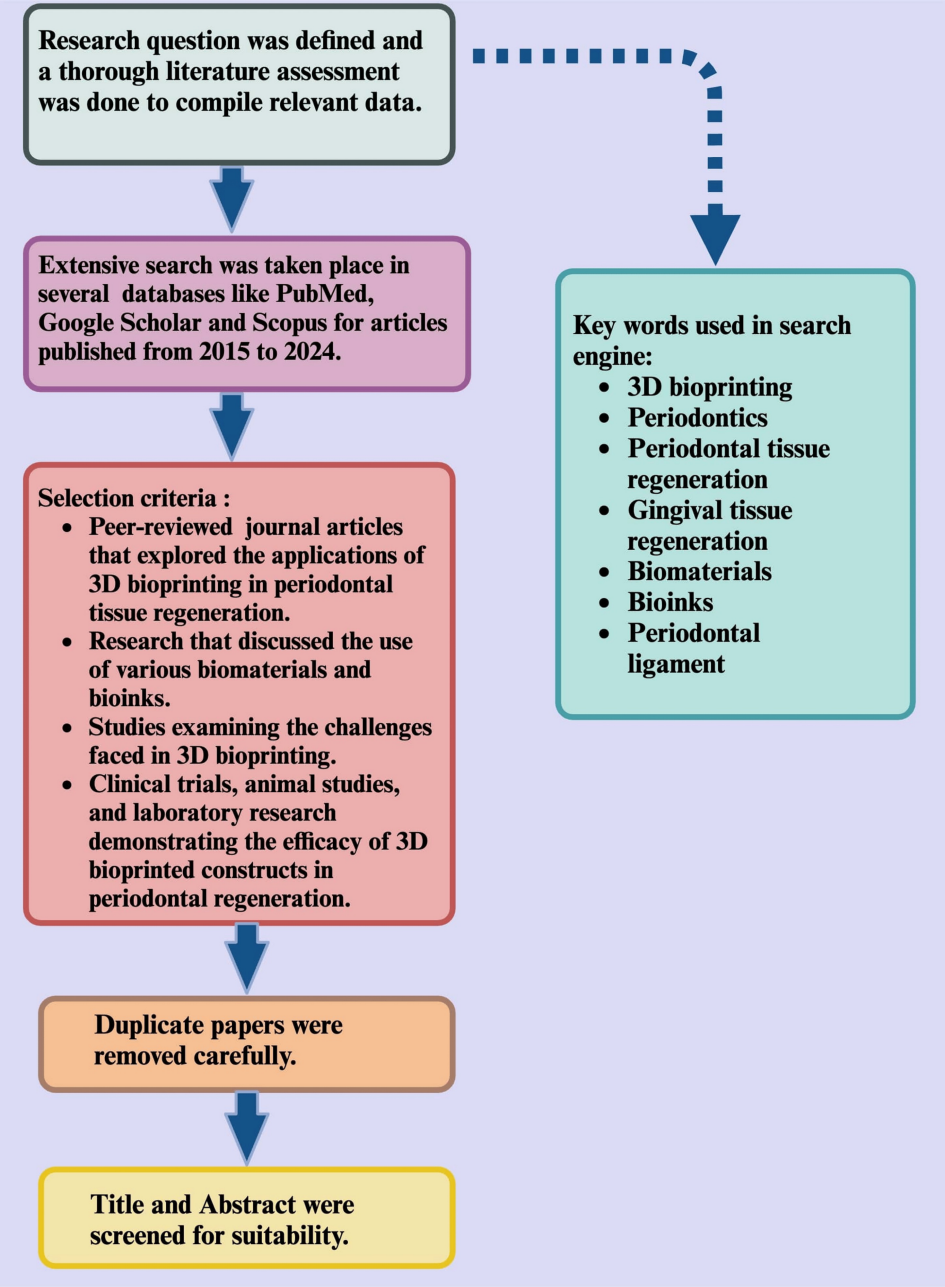
Methodology of the study. Illustration Credit: Dr. Jahnavi Acharya. This image was created using the premium version of BioRender [15] (https://BioRender.com/), accessed on February 22, 2025, with agreement license number ZN28164IZK. We did not utilize anything from the BioRender Template Library.

This review focuses on 3D bioprinting in periodontics, particularly for periodontal tissue regeneration. Relevant articles and studies were identified through comprehensive searches in several scientific databases, including Google Scholar, Scopus, and PubMed. Studies published between 2015 and 2024 were included in this review. The search terms utilized included "3D bioprinting", AND "Periodontics", AND "periodontal tissue regeneration", AND "Gingival tissue regeneration", AND "biomaterials", AND "bio-inks", AND "periodontal ligament", among others (Figure 3).

The selection criteria included the following: (i) peer-reviewed journal articles that explored the applications of 3D bioprinting in periodontal tissue regeneration focused on the PDL, alveolar bone, and gingival tissues; (ii) research that discussed the use of various biomaterials and bio-inks in the bioprinting of periodontal tissues; (iii) studies that examined the challenges faced in 3D bioprinting, such as vascularization, cell differentiation, and integration with host tissues; and (iv) clinical trials, animal studies, and laboratory research demonstrating the efficacy of 3D-bioprinted constructs in periodontal regeneration. This review synthesizes findings from these studies to provide a thorough understanding of the advancements and challenges, along with potential future directions of 3D bioprinting in periodontics.

Review of literature

3D bioprinting has emerged as a groundbreaking technology in regenerative medicine, including its application in periodontics. Traditional periodontal treatments, such as scaling and root planing, bone grafting, and soft tissue augmentation, have proven helpful in managing periodontal diseases. However, they often fail to restore the complex, functional architecture of the periodontal tissues, including the PDL, alveolar bone, and gingival tissues. 3D bioprinting technology in periodontal tissue regeneration has shown promise by enabling precise, personalized, and functional tissue constructs that restore these vital structures to their native state.

3D bioprinting techniques in periodontics

**Figure 4:**
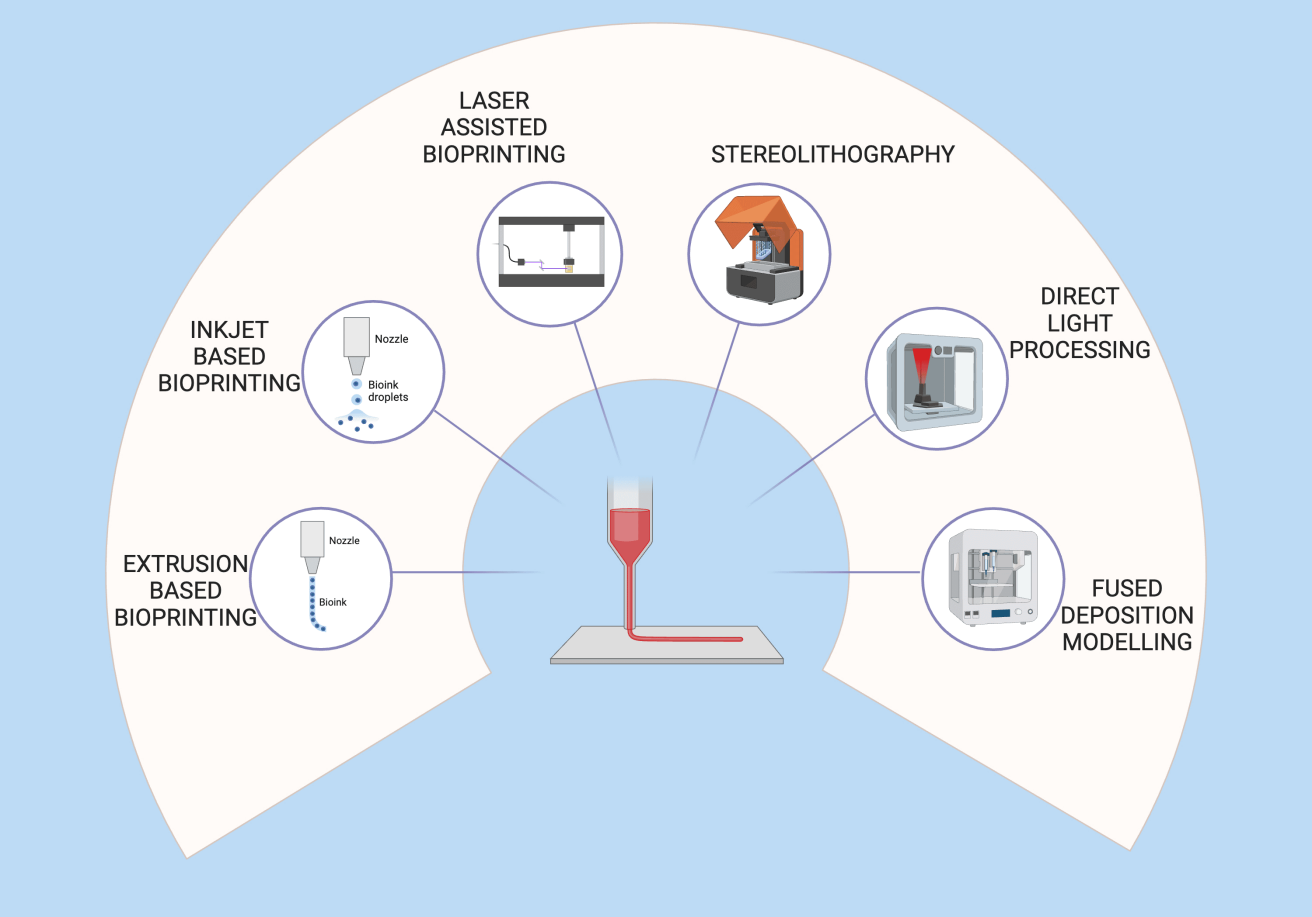
Methods of 3D bioprinting. Illustration Credit: Dr. Jahnavi Acharya. This image was created using the premium version of BioRender [15] (https://BioRender.com/487skxg), accessed on March 30, 2025, with agreement license number IB28367MB7. We did not utilize anything from the BioRender Template Library.

**Figure 5:**
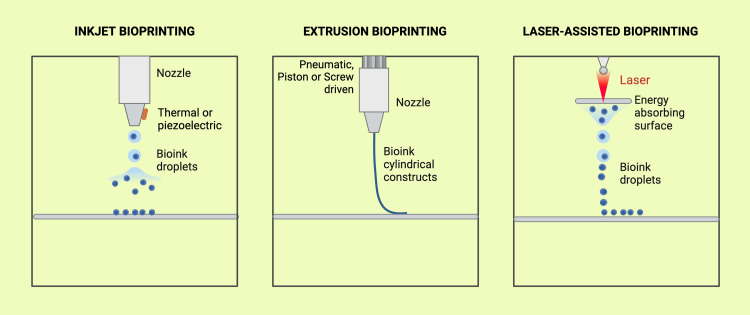
Three main types of 3D bioprinting processes used in periodontics. Illustration Credit: Dr. Jahnavi Acharya. This image was created using the premium version of BioRender [15] (https://BioRender.com/e08d892), accessed on March 30, 2025, with agreement license number NQ28362SPI. We did not utilize anything from the BioRender Template Library.

Although the core technology behind 3D printers remains the same, an automated, additive manufacturing process, different principles guide their operation. The technologies being considered include direct light processing (DLP), inkjet powder printing, fused deposition modeling (FDM), selective laser sintering (SLS)/direct metal laser sintering (DMLS), and stereolithography (SLA) (Figure 4) [31]. Although various bioprinting techniques have been developed, the most commonly used bioprinting technologies are extrusion, inkjet, and laser-assisted bioprinting (LAB) (Figure 5) [5].

Inkjet-Based Bioprinting

Inkjet-based bioprinting was the first approach to bioprinting. In this method, data from a computer is sent to the printer, which then reproduces it onto a substrate using ink droplets in a non-contact manner [32]. Three types of inkjet printers are used in this technique: thermal, piezoelectric, and mechanical. The printer cartridge is filled with bio-ink, which is pushed through a microfluidic reservoir and out of the nozzle. Initially, a considerable challenge in this process was cell death during printing caused by the rapid drying of the substrate. This issue was addressed by encapsulating the cells in highly hydrated polymers or hydrogels. In thermal inkjet printers, an electrical heat source warms the printhead, creating pressure that forces the bio-ink out of the nozzle [33]. In piezoelectric inkjet printers, applying voltage to the piezoelectric material causes it to change shape and produce acoustic waves that push the bio-ink into droplets at set intervals [34]. In mechanical inkjet printers, pressure is applied to force the bio-ink through the nozzle [35].

Laser-Assisted Bioprinting (LAB)

It is a technique where a laser is employed to deposit bio-ink onto a substrate. In this process, laser pulses are directed through a bio-ink-filled "ribbon" supported by a titanium or gold layer that absorbs and transfers energy to the ribbon [36]. The bio-ink and cells are suspended at the ribbon's bottom. When the laser pulse vaporizes the material, it creates a high-pressure bubble that pushes the biomaterial onto the substrate. LAB is a scaffold-free technique that enables high-resolution deposition of biomaterials. Since it does not use nozzles, it eliminates the risk of biomaterial clogging and can accommodate bio-inks of various viscosities. However, a significant drawback of LAB is the potential for metallic residues from the absorbing layers to remain on the printed structure, and the technology itself can be costly [37].

Extrusion-Based Bioprinting

**Figure 6:**
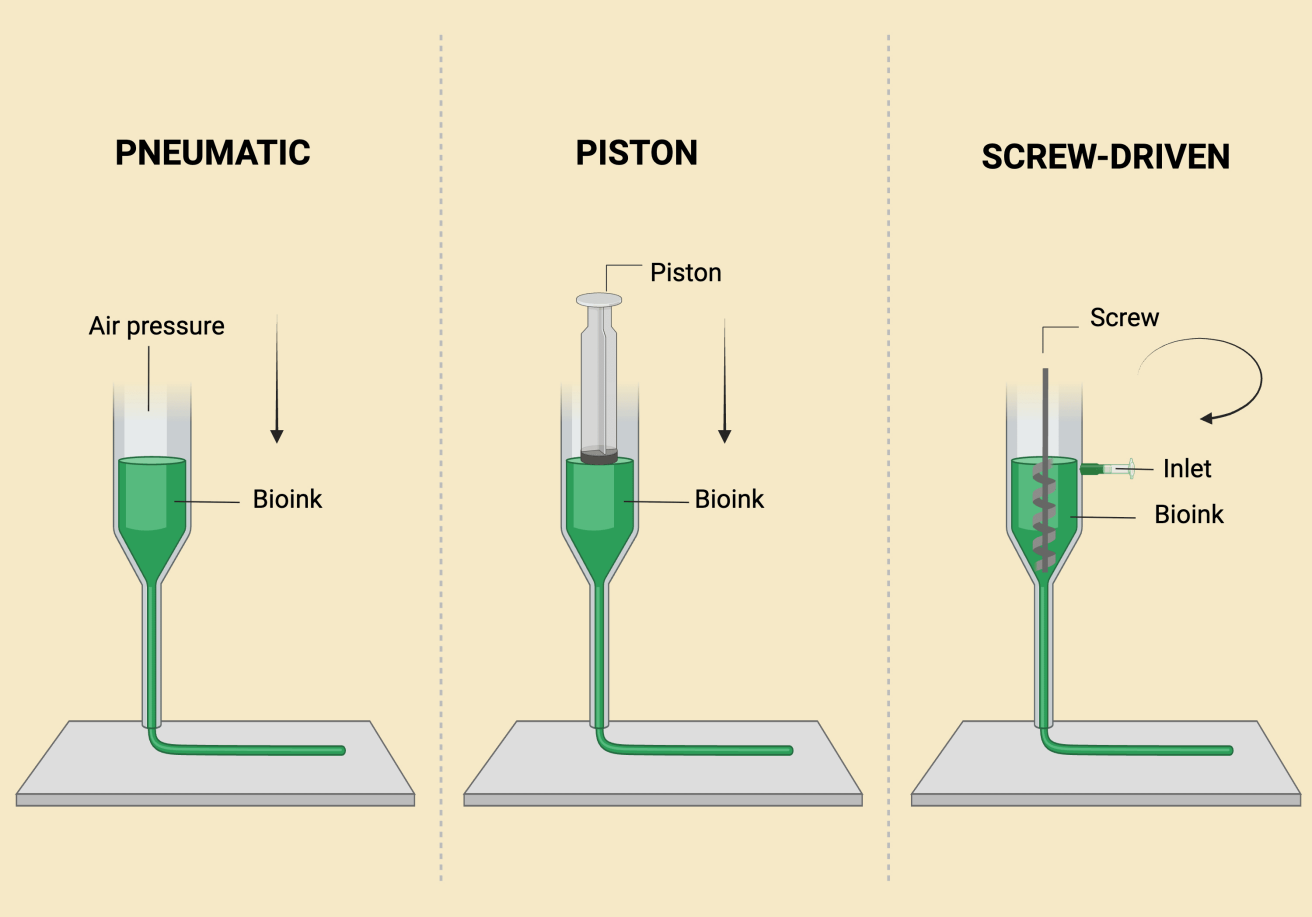
Extrusion-based bioprinting with three different mechanisms: pneumatic, piston-based, and screw-based. Illustration Credit: Dr. Jahnavi Acharya. This image was created using the premium version of BioRender [15] (https://BioRender.com/9xhw20i), accessed on March 30, 2025, with the agreement license number XW283GFBUZ. We did not utilize anything from the BioRender Template Library.

The printer has a fluid-dispensing system and an automated robotic system that extrudes the liquid to bioprint the structure. The system can be powered by a pneumatic, screw-driven, or piston-based system (Figure 6). The piston and screw-driven systems create the pressure necessary to eject the bio-ink, while the pneumatic system relies on pressurized air for extrusion [38]. This technique holds significant promise for creating biomimetic structures [39]. A key advantage is its ability to print with bio-inks that have high cell densities [40]. However, it has some limitations, including reduced resolution and the need for high pressure to extrude low-viscosity bio-inks, which may cause cell damage [41]. Extrusion-based printing systems are the most commonly used bioprinting technology in periodontal tissue engineering due to their ability to print the high-viscosity bio-inks necessary for supporting tissue scaffolds [24]. The versatility of extrusion bioprinting allows the incorporation of multiple cell types, such as fibroblasts, osteoblasts, and stem cells, essential for regenerating soft and hard periodontal tissues [26]. Other techniques, such as inkjet printing and LAB, are also being explored, though extrusion bioprinting remains the preferred method for periodontal applications [5].

Bio-inks

Bioprinting, the process of printing living tissues, relies on a specialized material called bio-ink. Bio-inks are crucial for this process and must exhibit several vital characteristics: they must be biocompatible, non-toxic, printable, capable of withstanding mechanical stresses, have good shape memory, and support cell nourishment while promoting cellular metabolic activities [42]. Bio-inks are typically composed of natural polymers, synthetic polymers, or a combination of both. To maintain the proper function of the living cells during 3D printing, a specific aqueous environment is necessary. This environment must regulate the correct pH, supply essential nutrients and oxygen, form an extracellular matrix, and ensure a non-toxic atmosphere to support cells forming new tissue. Hydrogels derived from extracellular matrix components like collagen and hyaluronic acid create this environment and promote stem cell growth [43]. However, since hydrogels are in a liquid polymer state, they are not strong enough to support multiple layers of cells during the printing process. To address these challenges, newer techniques, such as supramolecular bio-inks, thermoplastic reinforcement, nanocomposites, interpenetrating networks, and polymer functionalization, are employed to enhance the strength of hydrogels [44]. Bio-inks made from natural and synthetic materials, such as hydroxyapatite, alginate, and collagen, have been used in periodontal bioprinting due to their biocompatibility, ability to support cell growth, and potential to mimic the physical properties of native tissues [29,30].

Steps in 3D bioprinting

**Figure 7:**
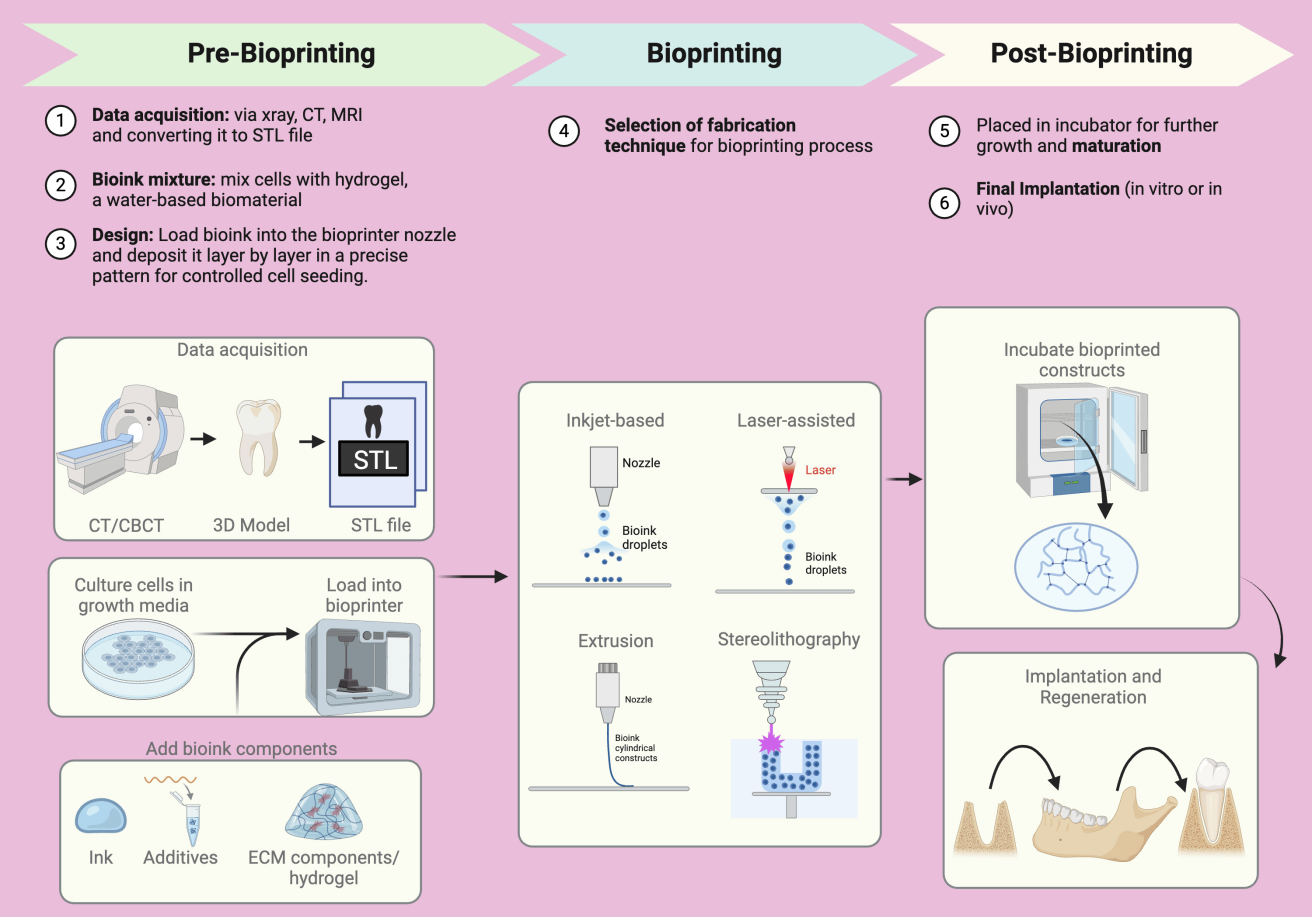
Steps in 3D bioprinting. CT: computed tomography, CBCT: cone beam computed tomography, MRI: magnetic resonance imaging, ECM: extracellular matrix, STL file: stereolithography (common file format used for 3D bioprinting/printing and computer-aided design representing objects in three dimensions). Illustration Credit: Dr. Jahnavi Acharya. This image was created using the premium version of BioRender [15] (https://BioRender.com/45kccut), accessed on March 30, 2025, with agreement license number NQ283GI1TD. We did not utilize anything from the BioRender Template Library.

The 3D bioprinting process consists of six key stages (Figure 7).

Data Acquisition

This stage involves obtaining data using techniques such as X-ray scanning, computed tomography (CT), magnetic resonance imaging (MRI), or directly from CAD software. The data is processed using specific software and converted into a printer-readable format [45]. The file is adjusted to estimate the amount of material to be extruded based on each layer's required height and width, which is influenced by the bio-ink's shape (droplet or filament) [46,47].

Selection of Bio-ink

Bio-ink is selected based on the printing technique and the specific needs of the printed structure. It must meet favorable mechanical properties, biocompatibility, and printability criteria. Bio-ink can also contain isolated cells, growth factors, and other bioprinting materials and should be prepared according to the printed structure's physiological temperature, pH, and requirements [46].

Setting Printing Parameters

This step involves adjusting the printing parameters, which depend on the chosen bio-ink and the desired properties of the final printed structure.

Actual Bioprinting

The printing process is closely monitored to allow for necessary adjustments. The printer and bio-ink type determine the printing resolution. For high-resolution prints, the fabrication time may be longer [48].

Post-printing Stage

The object may undergo spinning and microscopic evaluation after printing. It is then placed in an incubator or bioreactor to support further growth or maturation [29].

Placement of the Bioprinted Product

The final product is placed in either in vivo or in vitro conditions, depending on the intended application [29].

Applications in periodontal tissue regeneration

The main applications of 3D bioprinting in periodontics focus on the regeneration of the PDL, alveolar bone, and gingival tissues. These tissues have distinct structural and functional requirements, challenging their regeneration.

PDL Regeneration

The PDL is a soft connective tissue that plays a critical role in the attachment of teeth to the alveolar bone. In cases of periodontal disease, the PDL is often damaged, and its regeneration is a significant challenge. Researchers have used 3D bioprinting to create scaffolds that mimic the native PDL, incorporating cells like MSCs known to have regenerative potential. These MSCs, when combined with extracellular matrix (ECM) materials such as collagen, have demonstrated the ability to differentiate into functional PDL cells [24,29]. Studies have also explored using growth factors such as TGF-β to enhance PDL regeneration further and promote cell migration and differentiation [49].

Bone Regeneration

Alveolar bone loss is a frequent consequence of periodontal diseases, and its restoration is crucial for maintaining tooth stability. 3D bioprinting has created functional bone scaffolds that integrate with the host bone. Hydroxyapatite and calcium phosphate, which are biocompatible and osteoconductive, are often combined with osteoblasts or MSCs to promote bone formation. Recent studies have highlighted the ability of 3D-bioprinted bone scaffolds to support bone regeneration and reduce the need for traditional bone grafting [5,49]. Additionally, vascularization strategies, such as endothelial cells or angiogenic factors, are critical to ensuring the survival of sizeable bioprinted bone constructs [26,50].

Gingival Tissue Regeneration

Gingival recession is a typical result of periodontal disease, and it is essential to restore the gingival tissue to protect the underlying structures and improve aesthetic outcomes. 3D bioprinting has been explored for creating gingival tissue constructs using gingival fibroblasts combined with collagen and other ECM proteins. These bioprinted gingival constructs have shown the potential to promote healing and enhance tissue integration [29,51]. Further research on growth factors such as vascular endothelial growth factor (VEGF) aims to improve the vascularization of bioprinted gingival tissues, ensuring that the tissue survives and integrates into the host [52].

Challenges and advances in bioprinting for periodontics

While 3D bioprinting holds tremendous promise for periodontal tissue regeneration, several challenges must be addressed. 

Cell Viability and Differentiation

One of the foremost challenges is maintaining cell viability during bioprinting. The shear stress and temperature fluctuations associated with the printing process can damage cells and reduce their ability to differentiate into functional tissue types. To overcome this, researchers have developed advanced bio-inks that better protect cells during printing [53]. In addition, optimizing printing parameters, such as nozzle temperature and printing speed, is essential to ensure the highest cell viability [24].

Vascularization

The regeneration of larger tissues, such as the bone and gingiva, requires adequate vascularization to provide nutrient and oxygen supply. Currently, most bioprinted tissues are limited by the inability to form functional blood vessels within the printed construct. Strategies to overcome this limitation include incorporating endothelial cells, angiogenic growth factors like VEGF, and co-culture techniques to promote vascularization [5,26]. The development of functional vascular networks within bioprinted tissues remains a critical area of research.

Tissue Integration

Integrating bioprinted tissues with host tissues is essential for long-term functional outcomes. Studies have demonstrated the ability of 3D-bioprinted bone constructs to integrate with host bone, but further research is needed to understand the mechanisms behind tissue integration, particularly for soft tissues like the PDL and gingiva [54]. Additionally, the mechanical properties of bioprinted constructs must closely match those of the native tissues to ensure proper integration and function.

Prospective advantages and mode of action of 3D bioprinting in periodontics

Table 1 highlights the prospective advantages and modes of action of 3D bioprinting in periodontics, emphasizing how this technology can revolutionize periodontal tissue regeneration.

**Table 1:** Advantages and mode of action of 3D bioprinting in periodontics. CT: computed tomography, MRI: magnetic resonance imaging, TGF-β: transforming growth factor-beta, VEGF: vascular endothelial growth factor. Table Credit: Dr. Jahnavi Acharya.

Advantages and mode of action	Explanation
Precision in tissue architecture	3D bioprinting allows for the exact layer-by-layer deposition of bio-inks, which can precisely replicate the complex structural and functional properties of periodontal tissues. This precision helps create tissue constructs that match the native architecture of the periodontal ligament (PDL), alveolar bone, and gingiva, ensuring optimal integration with the host tissue [24]
Personalized and patient-specific constructs	One of the most critical aspects of 3D bioprinting is the ability to create customized, patient-specific tissue constructs. By utilizing patient-derived imaging data, such as from CT or MRI scans, bioprinting can tailor tissue constructs that address each patient's unique anatomical and pathological needs, offering improved treatment outcomes [29]
Cell and growth factor delivery	Bio-inks containing stem cells, growth factors (e.g., TGF-β for PDL regeneration), and other signaling molecules can be precisely incorporated into 3D-bioprinted constructs. This allows for the controlled release of bioactive molecules to promote cellular differentiation, migration, and regeneration in periodontal tissues [26]
Regeneration of hard and soft tissues	3D bioprinting facilitates the regeneration of both hard tissues (e.g., alveolar bone) and soft tissues (e.g., gingiva, periodontal ligament) by using biomaterials that mimic the mechanical and biological properties of native tissues. This ability to regenerate both tissue types is beneficial in treating the multi-tissue nature of periodontal disease [55]
Enhancement of vascularization	One of the key challenges in tissue engineering is ensuring sufficient vascularization for nutrient and oxygen supply, particularly in larger tissue constructs. 3D bioprinting can incorporate endothelial cells and angiogenic growth factors like VEGF, promoting the formation of capillary networks within bioprinted tissues, thus ensuring their survival post-implantation [5]
Improved integration with host tissues	The precise printing of bio-inks replicating the extracellular matrix (ECM) allows for better cell adhesion, migration, and integration with the host tissue. This is critical for the long-term functionality and stability of the bioprinted tissue once implanted [56]
Reduction of invasive surgeries	3D bioprinting can reduce the need for traditional, invasive surgical interventions such as bone grafting and soft tissue augmentation. By bioprinting the required tissues in a laboratory and implanting them directly into the defect sites, patients could experience faster recovery times and fewer complications [28]
Optimized scaffold design for tissue growth	3D-bioprinted scaffolds can be designed to support tissue growth with the ideal porosity, mechanical strength, and surface features. This promotes better tissue formation and structural support, particularly in complex periodontal tissues like the PDL and alveolar bone [26]
Reduction of immune rejection	Using autologous cells (cells from the patient's tissue) in bio-inks minimizes the likelihood of immune rejection, improving the chances of success for bioprinted implants and grafts in periodontal treatment [30]

Research advances in 3D bioprinting for periodontics

Table 2 presents findings from numerous studies on using 3D bioprinting in periodontics, highlighting key tissue types, materials, and outcomes. The studies primarily focus on regenerating periodontal tissues like the PDL, alveolar bone, and gingiva, using a combination of bio-inks, stem cells, and growth factors. Each study presents a unique approach, exploring the effects of different bio-inks (e.g., collagen, hydroxyapatite, and polycaprolactone (PCL)), stem cells (e.g., MSCs), and growth factors (e.g., TGF-β, VEGF, and platelet-derived growth factor (PDGF)) on the regeneration process. 

**Table 2:** Findings regarding 3D bioprinting in periodontics. MSCs: mesenchymal stem cells, VEGF: vascular endothelial growth factor, BMP2: bone morphogenic protein 2, PDGF: platelet-derived growth factor. Table credit: Dr. Jahnavi Acharya.

Study/research	Findings	Tissues regenerated	Materials used	Key outcomes
Vijayavenkataraman et al. [24]	Explored the potential of 3D bioprinting for creating periodontal tissues, including the PDL and alveolar bone, using MSCs and collagen-based bio-inks	PDL, alveolar bone	Collagen, hydroxyapatite, MSCs	Successful PDL formation with MSCs; enhanced bone regeneration with hydroxyapatite
Gul et al. [30]	Investigated the role of 3D printing in periodontal tissue regeneration, focusing on creating scaffolds for bone and soft tissue regeneration	Bone, gingiva	Polycaprolactone (PCL), collagen	Improved scaffold architecture promoting cell adhesion and tissue integration
Almeida et al. [26]	Examined the use of bio-inks for periodontal tissue regeneration, mainly focusing on bio-ink formulations for PDL and gingival tissue regeneration	PDL, gingiva	Alginate, gelatin, MSCs, collagen	Enhanced tissue maturation and cell proliferation with custom bio-inks
Zhao et al. [28]	Focused on bioprinting alveolar bone structures and integrating stem cells for osteogenesis, emphasizing creating patient-specific constructs	Alveolar bone	Hydroxyapatite, MSCs	Increased osteogenic differentiation and bone formation when combined with MSCs
Ostrovidov et al. [5]	Investigated bioprinted alveolar tissue regeneration, utilizing endothelial cells and growth factors for angiogenesis in bone constructs	Alveolar bone, vascularization	Hydroxyapatite, endothelial cells, VEGF	Successful vascularization of printed bone constructs; enhanced osteointegration
Sufaru et al. [29]	Explored the development of bioprinted membranes and scaffolds for periodontal regeneration, focusing on the regeneration of gingival tissues using bio-inks	Gingiva, soft tissue	Collagen, fibrinogen, MSCs	Gingival tissue regeneration with faster healing and improved integration with the host tissue
Liu et al. [57]	Focused on developing bio-inks for the 3D bioprinting of gingival tissues, highlighting the role of fibroblasts and collagen in tissue formation	Gingiva	Collagen, fibroblasts	Fibroblast-laden bio-inks promoted healthy gingival tissue formation with better structural integrity
Miao et al. [58]	Studied incorporating stem cells and growth factors in bioprinted scaffolds for regenerating alveolar bone and gingival tissues	Alveolar bone, gingiva, PDL	Gelatin, sodium alginate (SA), bioactive glass microspheres (BGM)​, BMP2, PDGF	Enhanced bone regeneration and soft tissue healing demonstrate combined regeneration potential

For instance, Vijayavenkataraman et al. [24] and Sufaru et al. [29] found that using MSCs in collagen and other ECM-based bio-inks promoted successful PDL and gingival regenerations. Similarly, Zhao et al. [28] and Ostrovidov et al. [5] emphasized the importance of incorporating hydroxyapatite for bone regeneration and endothelial cells for vascularization in bioprinted bone constructs. Studies like that of Miao et al. [58] explored the combination of stem cells and growth factors to promote the regeneration of both bone and gingival tissues. Overall, the findings from these studies indicate that 3D bioprinting holds significant potential for advancing periodontal tissue regeneration, with successful outcomes depending on the combination of appropriate biomaterials, stem cells, and growth factors to support tissue integration and healing.

Limitations of the 3D bioprinting technique

The study on 3D bioprinting in periodontics presents numerous promising outcomes, but several limitations must be considered. These limitations highlight the challenges in translating the technology from the laboratory to clinical practice. Below are some of the key limitations of the study.

Limited Long-Term Clinical Data

Although assorted studies have demonstrated promising results in animal models and in vitro, long-term clinical data is lacking to confirm the durability and effectiveness of 3D-bioprinted periodontal tissues in human patients. Most studies are still in the preliminary stages, and more extensive clinical trials are needed to assess the long-term success of bioprinted constructs in periodontal regeneration [59].

Vascularization Challenges

A significant limitation of current 3D bioprinting technology is the difficulty in creating large, fully functional vascular networks within bioprinted constructs. The inability to ensure proper vascularization hinders the regeneration of larger tissues, such as alveolar bone and gingiva: bioprinted tissues risk necrosis or poor integration with host tissues without an adequate blood supply. While advancements in angiogenesis are underway, achieving complete vascularization remains a challenge [5,26].

Complex Multi-tissue Regeneration

The regeneration of the periodontium involves the coordination of multiple tissue types (bone, soft tissue, PDL) with distinct mechanical and biological properties. While 3D bioprinting has shown potential in regenerating individual tissues like PDL or alveolar bone, achieving simultaneous and successful regeneration of all periodontal tissues within a single construct remains difficult. The complexity of the tissue interactions and the need for precise cellular organization further complicate this task [29].

Limited Biomaterial Options

Though various biomaterials such as collagen, hydroxyapatite, and PCL are used in 3D bioprinting, the range of materials that can accurately replicate natural periodontal tissues' mechanical and biological properties is still limited [60]. Additionally, many materials face challenges in terms of long-term stability and degradation rates. New bio-inks must be developed to mimic the native ECM of periodontal tissues to promote more effective tissue regeneration [30].

Cell Viability and Differentiation

Another challenge in 3D bioprinting is maintaining cell viability throughout the printing process. The shear forces involved in extrusion-based printing can damage delicate cells, affecting their ability to proliferate, differentiate, and integrate with the surrounding tissues. Although recent advancements in bio-ink development have helped mitigate these issues, ensuring cells' sustained viability and functionality remains a significant hurdle [54].

Scalability and Cost

While 3D bioprinting shows enormous potential, it is still a relatively expensive and labor-intensive process. Scaling up the production of bioprinted tissues for clinical use is challenging due to the prohibitive costs of specialized equipment, bio-inks, and the time-consuming nature of the printing process. These factors limit the accessibility and affordability of 3D bioprinting technologies, especially in low-resource settings [24].

Regulatory and Ethical Challenges

As 3D-bioprinted tissues move closer to clinical application, regulatory and ethical considerations become more prominent. Using stem cells, bio-inks, and genetically modified cells in bioprinting raises ethical concerns about patient safety, consent, and potential long-term consequences. Additionally, regulatory agencies like the FDA must establish clear guidelines for approving bioprinted tissues, ensuring their safety and efficacy for human use [28].

Integration With Host Tissues

Even though 3D-bioprinted tissues show promise in experimental models, integrating these tissues with the host tissue in a clinical setting remains a significant challenge. The bioprinted tissues must mimic the structure and function of natural tissues and successfully integrate with the surrounding tissue environment to restore full functionality. Achieving this integration without rejection or adverse immune reactions is still a primary barrier to successful clinical application [61].

Future research perspective

The future of 3D bioprinting in periodontics holds excellent promise. As advancements in biomaterial science and bioprinting technologies continue, we can expect more sophisticated bio-inks that better mimic the native ECM of periodontal tissues [59]. In addition, integrating artificial intelligence (AI) and machine learning into bioprinting could create more precise, patient-specific tissue constructs. These technologies could optimize the design of bioprinted tissues by tailoring them to individual patient's needs, improving the success rates of regenerative therapies [5]. Moreover, with ongoing efforts to enhance vascularization and tissue integration, 3D bioprinting could provide a viable alternative to conventional grafting and tissue engineering techniques, offering more predictable and functional outcomes for periodontal patients [26,29]. However, for a comprehensive understanding of the clinical potential of 3D bioprinting, further research is required in areas such as the development of hybrid bio-ink, standardization of printing protocols, and long-term in vivo studies to assess the safety, functionality, and integration of printed constructs.

## Conclusions

While 3D bioprinting offers exciting possibilities for periodontal tissue regeneration, it is still in the developmental stages and faces several challenges. Addressing the limitations related to vascularization, multi-tissue regeneration, biomaterial development, and clinical validation will be crucial for successfully applying 3D bioprinting in periodontics. Future research and technological advancements must overcome these obstacles to ensure that 3D bioprinting can be used effectively and safely in clinical periodontal therapies. 
